# A systematic review of trends in photobiomodulation in dentistry between 2018 and 2022: advances and investigative agenda

**DOI:** 10.12688/f1000research.140950.1

**Published:** 2023-10-27

**Authors:** David Yeret Rodriguez Salazar, Jimmy Alain Málaga Rivera, José Edinson Laynes Effio, Alejandro Valencia-Arias

**Affiliations:** 1 Universidad Senor de Sipan, Chiclayo, Lambayeque, Peru; 2 Universidad Peruana de Ciencias Aplicadas, Lima District, Lima Region, Peru; 3 Universidad Nacional Pedro Ruiz Gallo, Lambayeque, Lambayeque, Peru

**Keywords:** photomodulation, PBM, wavelength, light amplification, Laser Therapy, Medical sciences, Dentistry, Dentistry, Esthetic Dentistry

## Abstract

**Background:**

Photobiomodulation (PBM) involves laser therapy utilized in medical sciences to modulate biological processes acting as a palliative and immune response-enhancing treatment. This study conducts a comprehensive bibliometric analysis to explore current trends in PBM-related scientific production, encompassing publications, citations, impact, keywords and clusters. Additionally, it aims to predict future research trends in this domain.

**Methods:**

The data for this quantitative and qualitative bibliometric analysis were obtained from 608 scientific documents retrieved in November 2022, with 123 sourced from Web of Science and 485 from Scopus, Utilizing Excel, the data was processed in Excel to extract essencial information. Productivity and impact were evaluated for eligibility, and VOSviewer aided in determining associativity for the bibliometric analysis.

**Results:**

The findings of this study demostrate that the scientific production related to PBM adheres to a growth power law, exhibiting characteristics of both exponential and linear phases. Notably, recent research trends emphasize critical concepts such as laser therapy, orthodontics, and dental pulp stem cells. Particularly significant is the burgeoning interest in utilizing PBM within dentistry as a complementary alternative to existing protocols.

**Conclusions:**

PBM stands as a promising laser therapy within medical applications. Through a detailed bibliometric analysis, this study underscores the increasing significance of PBM, especially within the realm of dental treatments. These insights offer a glimpse into the evolving landscape of PBM research and provide valuable guidance for potential future directions of study.

## Introduction

The main reason for seeking health care is pain, and currently, as an alternative treatment, laser light, known as PBM, has been established as an important noninvasive therapy. ^1^^,^^2^ PBM stimulates the healing and regenerative process, modifies certain harmful processes, ^3^ ameliorates inflammation and pain, and activates the immune response against pathogens. ^3^^–^^5^

Regarding analgesic therapy, PBM has actions at the levels of local and systemic pathways, favouring vasodilation, improving lymphatic drainage, generating axonal depolarization, and reducing vasoactive amines (prostaglandins - leukotrienes) and cytokines. ^6^

The subjective experience of pain ^7^ is associated with a delay in the wound healing process, which is affected by various causes, such as stress, psychological state and type of wound closure, among others. ^8^^,^^9^ PBM has an effect on the scarring process, but the parameters used must be taken into account to achieve an optimal dose that ensures the desired effect. ^10^

PBM, as a clinically noninvasive therapy, has been shown to exert beneficial effects in neurosensory recovery, in the restoration of functional disability, ^11^^,^^12^ in the treatment of musculoskeletal injuries, in degenerative diseases ^13^ and in the healing process, both in regenerative medicine and dentistry ^12^; however, to date, its use in dentistry has been limited. ^13^

In dentistry, PBM is used to generate, at the cellular level, an increase in differentiation and replication in alveolar bone and to biostimulate and regenerate soft tissues. ^14^ Correct wound healing and reducing the intensity and duration of postoperative pain consequently improve prognoses and result in periodontal treatment efficacy and patient comfort. ^15^

Additionally, regarding periodontal surgery, there are still controversies regarding the effect of PBM with respect to wound healing and reducing postoperative pain. ^16^^–^^18^

Therefore, the main objective of this article is to investigate the trends in the application of PBM between 2018 and 2022 using a bibliometric analysis of publications retrieved from Scopus and Web of Science. Specifically, this systematic review aims to:

‐ Analyze the trends in the use of PBM in dentistry between 2018 and 2022.
‐ Identify the key areas of application for PBM in dental practice.
‐ Evaluate the effects of PBM on wound healing and postoperative pain reduction in dentistry.
‐ Explore controversies and gaps in the current literature regarding the use of PBM in dentistry.

Among the conclusions, the main trends in the use of PBM are based on the biostimulation and regeneration of soft tissue and hard tissue, with a consequent decrease in inflammatory mediators, thus producing analgesia.

## Methods

To address the research objective, an exploratory bibliometric analysis was conducted to assess scientific activity in this field. ^19^ Additionally, the study was carried out following the parameters established by the PRISMA statement for conducting literature reviews. ^20^^,^^21^

### Eligibility criteria

Both inclusion and exclusion criteria were established for the study selection process. Inclusion criteria encompassed all articles that, in the main scientific metadata, such as title and keywords, include terms such as oral health and PBM, as well as their respective synonyms, validated by thesauri such as that of UNESCO.

Regarding the exclusion criteria, we followed two consecutive phases in accordance with the PRISMA statement. The first phase involved screening, which entailed the omission or exclusion of articles that exhibited indexing errors, as such publications do not allow the quantitative analysis of the main research metadata. Likewise, all records with themes that are different from the objective of the review are excluded.

The second phase of exclusion, referred to as eligibility, involved eliminating all publications, that, having passed the first phase of exclusion, that show evidence of insufficient methodological rigor are eliminated.

### Source of information

To obtain publications for the bibliometric analysis, the two main databases in terms of scientific coverage, rigor in evaluation processes, thematic diversity and obtaining metadata ^22^ were selected as sources of information: Scopus and Web of Science.

### Grouping for synthesis

The selected studies will be grouped into thematic categories based on their approaches and findings. This will facilitate a qualitative synthesis of findings related to the application of PBM in dentistry between 2018 and 2022. Furthermore, we will consider variability among the studies and explore potential subgroups for more detailed analyses.

### Search strategy

Once the source of information for the literature review process had been defined, a search strategy was devised for the specific search interface of each database considering the inclusion criteria, resulting in two specialized search queries. In Scopus, we implemented the following search strategy:

(TITLE ((dent* OR “oral health” OR bucal) AND (photobiomodulation* OR photomodulation* OR pbm OR wavelength OR “light amplification*”))) OR (KEY ((dent* OR “oral health” OR bucal) AND (photobiomodulation* OR photomodulation* OR pbm OR wavelength OR “light amplification*”))).

The search strategy for Web of Science mirrored that of Scopus in terms of terminology and metadata but adapted to the distinct search interface, resulting in the following search strategy:

(TI= ((dent* OR “oral health” OR bucal) AND (photobiomodulation* OR photomodulation* OR pbm OR wavelength OR “light amplification*”))) OR (AK= ((dent* OR “oral health” OR bucal) AND (photobiomodulation* OR photomodulation* OR pbm OR wavelength OR “light amplification*”))).

### Data management

The search strategies retrieved a total of 608 scientific documents, with 123 sourced from Web of Science and 485 from Scopus. These documents were stored and processed in Microsoft Excel®, during which all duplicate documents were eliminated. The two exclusion phases defined in the eligibility criteria were then applied. Additionally, bibliometric indicators, enabling the evaluation of productivity and impact of authors, journals, and countries ^23^ were extracted. The free access software VOSviewer was utilized to visualize associativity, scientific cooperation factors and thematic relationships.

### Methodological design

**Figure 1.**
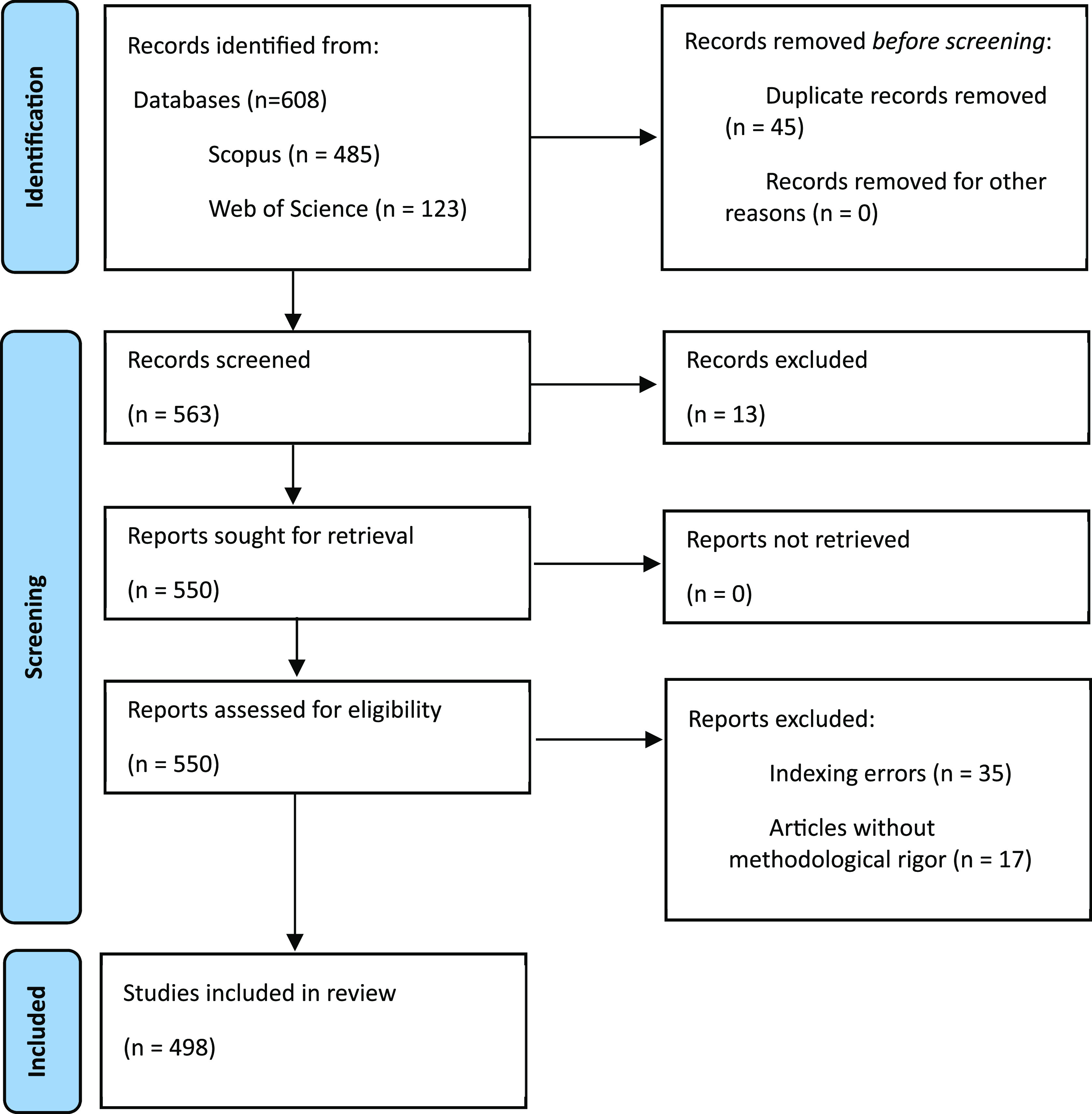
PRISMA flow chart for this literature review.

Finally, in accordance with the international PRISMA statement for literature review processes, we provide a flow diagram illustrating the methodological design in Figure 1.

As evidenced, publications are identified using search strategies in the two selected databases, and all identified duplicate articles were eliminated. Subsequently, two phases of exclusion, i.e., screening and eligibility, were applied. Ultimately, 498 documents were included in the bibliometric analysis.

## Results

### Publications per year

**Figure 2.**
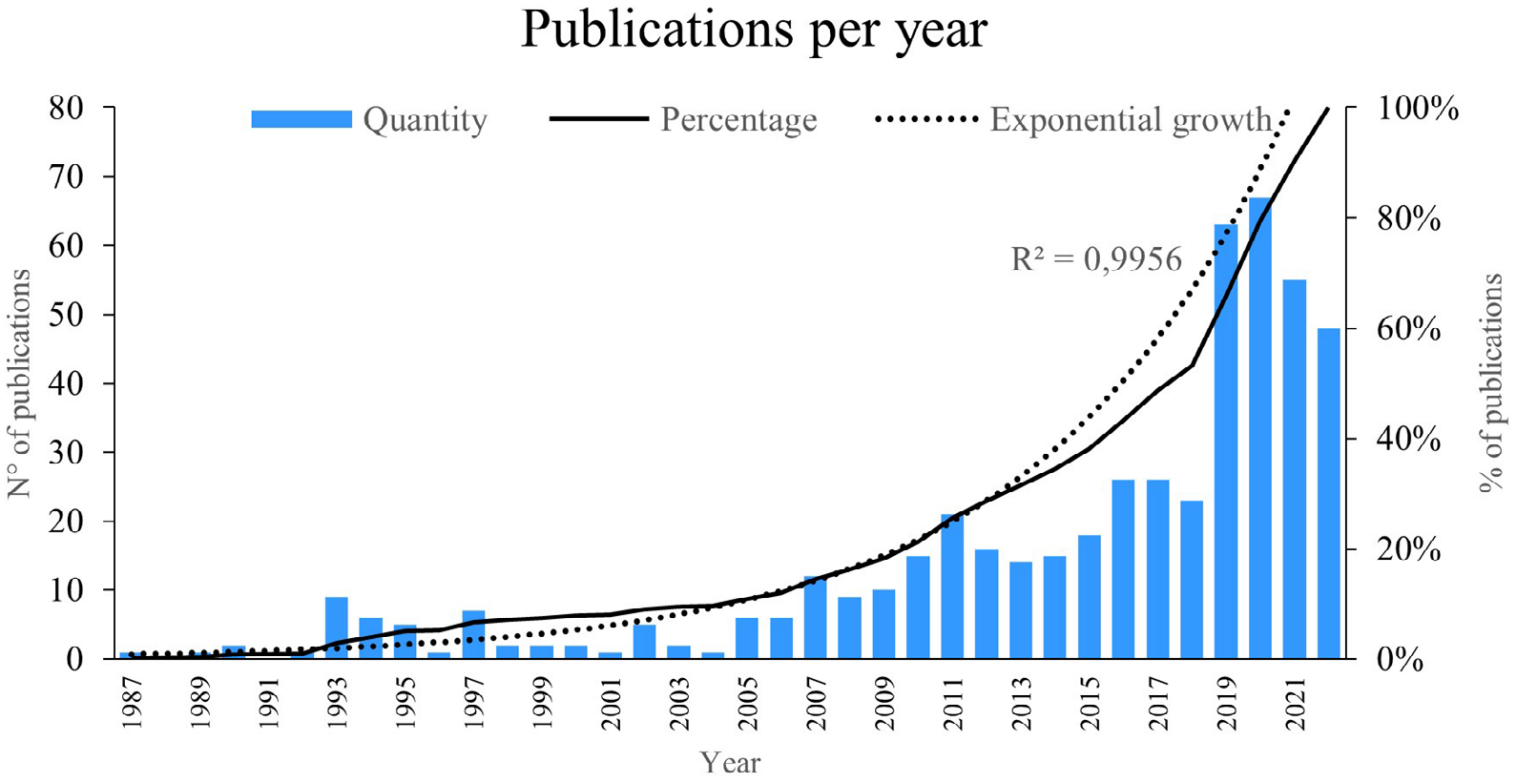
Publications per year.

The indicator “publications per year” reflects the number of new works published within a specific timeframe in a particular research field. Figure 2, we illustrate the number of studies published from 1987 to 2022, revealing an exponential growth of 99%. Notably, the years 2019 and 2020 saw the highest number of publications. Particularly, the year 2020 stood out with the most publications on PBM, reaching 67; some of these publications explored how laser therapy induces a photobiomodulatory effect in cells and tissues, contributing to improvements in reparative processes. ^24^

The year 2019 had the second highest scientific productivity on the subject, with a total of 63 publications; some of the articles investigated the way in which low-energy PBM therapy favours cell therapy by improving cell sheet transplantation. ^25^

### Publications per author

**Figure 3.**
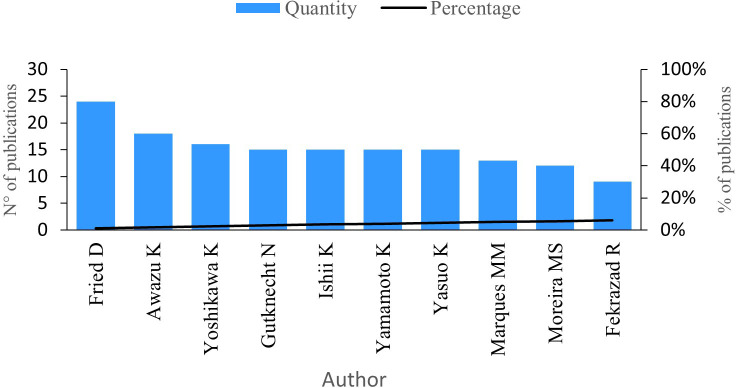
Publications per author.

The next indicator analysed is the number of publications by author. Figure 3 shows the 10 authors with the highest number of publications in the research field. Fried D, with a total of 24 publications, has investigated the development of clinical probes with the ability to acquire transillumination and infrared reflectance images with short wavelengths and the diagnosis of lesions on the occlusal tooth surfaces, among other lines of study. ^26^

Awazu K stands as the second most productive author in this research field, boasting 18 publications. His works delve into less invasive procedures utilizing pulsed nanosecond lasers, aiming to reduce tissue damage and enhance dental caries treatment. ^27^ Additionally, he has conducted research on the effects of lasers with a wavelength of 6.2 μm, specifically their absorption capabilities for dental caries without causing harm to dental tissue. ^28^

Furthermore, Yoshikawa K has contributed with 16 publications, followed by Gutknecht N, Ishii K, Yamamoto K, and Yasuo K, each boasting 15 publications. Marques MM follows closely with 13 publications, succeeded by Moreira MS with 12 publications and Fekrazad R with nine publications.

### Publications per journal

**Figure 4.**
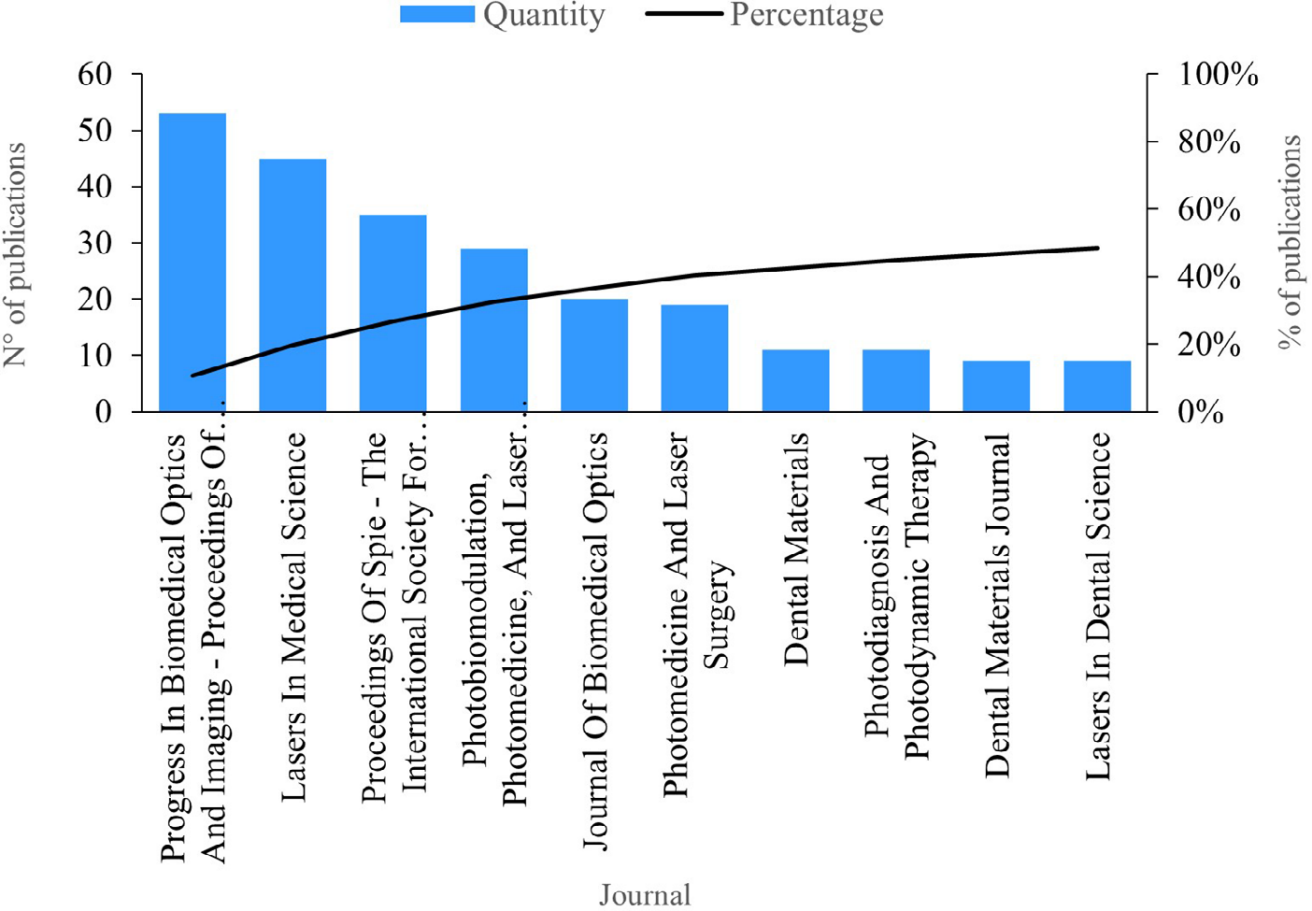
Publications per journal.

The indicator “publications per journal” signifies the number of publications within the field of study attributed to a scientific journal. In Figure 4, we present the top ten journals with the highest number of publications. Leading the productivity chart is the journal “Progress In Biomedical Optics And Imaging - Proceedings Of SPIE”, boasting 53 publications on PBM. Studies within this journal delve into various aspects, including the dehydration dynamics of fluorosis lesions, revealing its similarity to caries lesions. ^29^ Furthermore, these studies demonstrate the efficacy of SWIR light at 1950 nm, showcasing an exceptionally high demineralization contrast and its optimal use in assessing lesion activity on tooth surfaces. ^30^

Next, “Lasers in Medical Science” had 45 publications. Among the published studies, the efficacy of the Fenton reagent in the bleaching process was investigated, as well as its ability to improve the performance of bleaching agents when combined with light. ^31^ Additionally, a study explored the positive bioenergetic effects of PBM on the mitochondria of osteoblasts among dental pulp stem cells in humans. ^32^

### Publications per country

**Figure 5.**
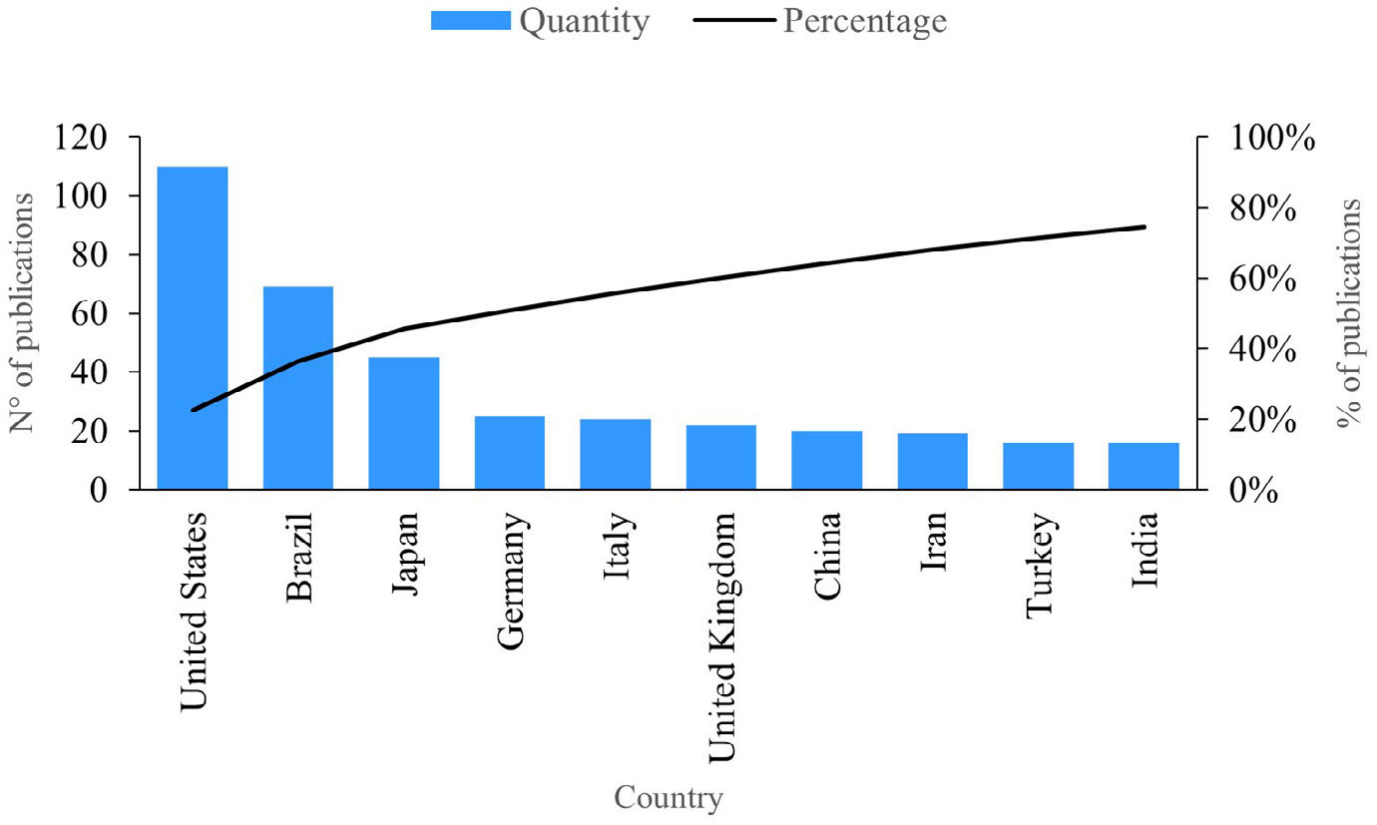
Publications per country.

This indicator represents the trends for countries in terms of publications pertaining to PBM. Figure 5 presents the ten countries with the highest level of productivity in the field of research. The first is the United States, with 110 publications, including one that shows how biophotonic approaches can reduce the burden of microorganisms, decontaminate surfaces and tissues, and avoid the spread of viruses through minimally invasive techniques ^33^ and one that investigate the clinical efficacy and safety of photon energy transfer during PBM dosing. ^34^

Brazil has 69 publications on the subject. In these studies, authors demonstrate how PBM utilizing light-emitting diode (LEDs) can be effectively combined with biomaterials to promote bone formation, control pain, and the manage the inflammatory process. ^35^ Additionally, these studies identify that irradiation strategies employing red LEDs proved to be effective in reducing concentrations of nitric oxide (NO) and reactive oxygen species (ROS), while also stimulating the viability of human dental pulp fibroblasts exposed to lipopolysaccharides. ^36^

### Citations per author

**Figure 6.**
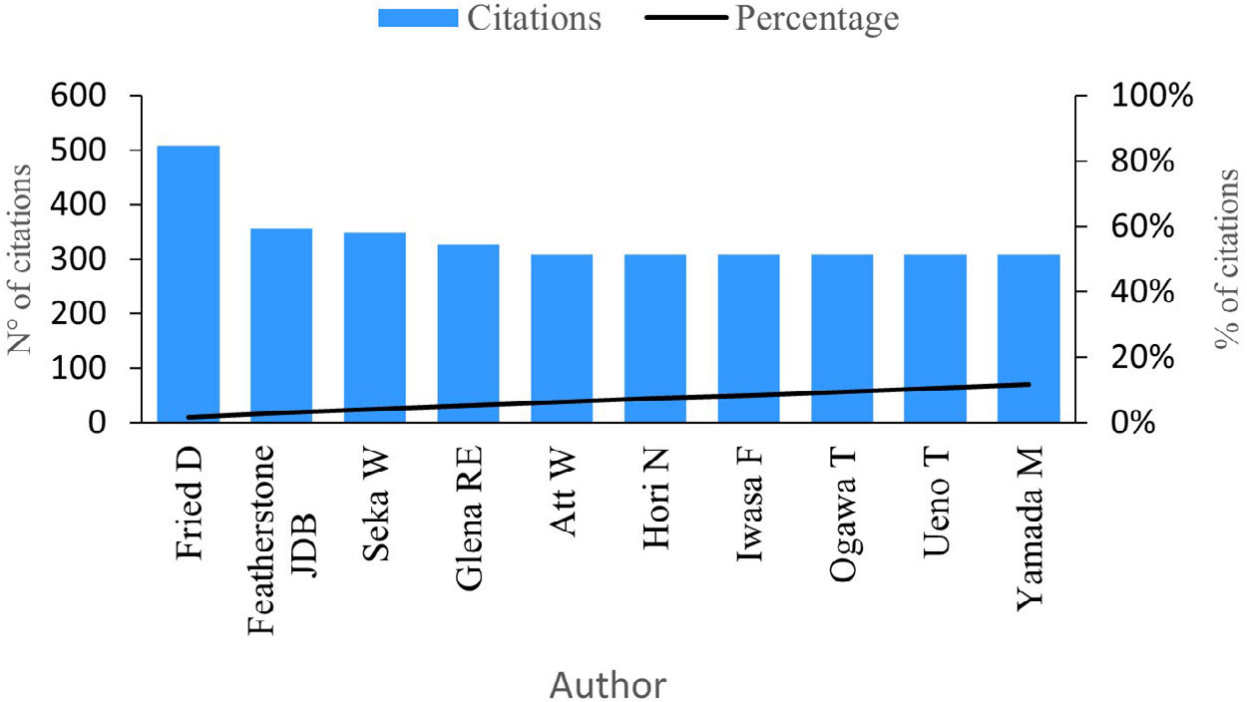
Impact per author.

This indicator gauges the impact authors have made by considering the number of citations linked to their research work. Figure 6 showcases the top ten authors with the highest number of citations in the research field. At the forefront is Fried D with an impressive 508 citations for his 24 publications. Notably, Fried D holds the title for the author with the most significant scientific impact, making him a pivotal reference in the research field (see Figure 3). One of his most cited articles, focusing on the nature of light scattering concerning dental enamel and dentin through a comparison of scattering data using Monte Carlo scattering simulators with angular resolution, ^37^ has been cited in 327 publications.

Featherstone J has garnered an impressive 357 citations for his contributions across five publications on PBM. His analysis delves into the measurements of the inhibition of dental caries subsequent to enamel irradiation. Particularly, Featherstone’s research reveals that enamel conditioned with a laser exhibits a more resistant surface to acid dissolution compared to untreated enamel. ^38^

### Citations per journal

**Figure 7.**
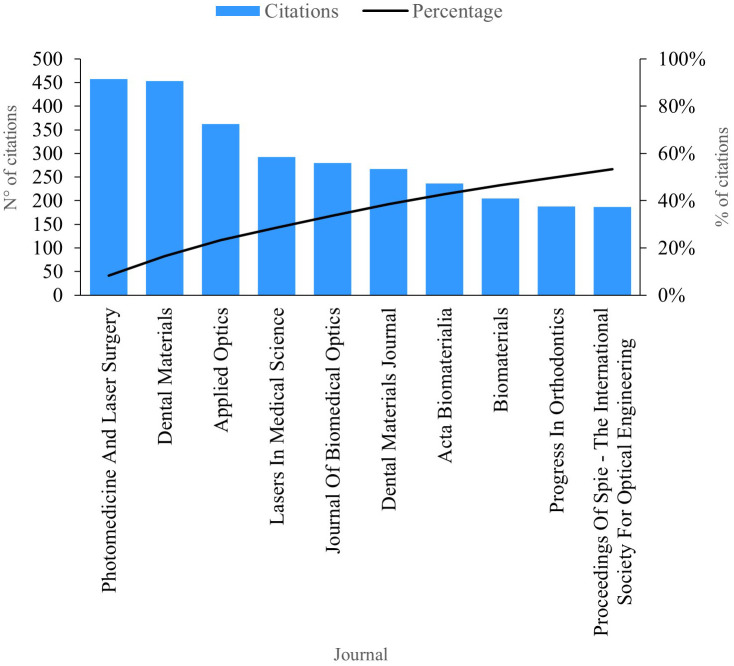
Impact per journal.

This review of scientific literature on PBM enabled the identification of the ten journals currently boasting the greatest scientific impact based on the number of citations, as depicted in Figure 7. “Photomedicine And Laser Surgery”, amassing a total of 457 citations and holding the mantle of the most productive journal in this domain. Publications within this journal extensively analyze the bacterial efficacy of antimicrobial photodynamic therapy as a complement to scraping and radicular smoothing in periodontal disease. ^39^

“Dental Materials” stands out with an impressive 453 citations. The publications in this esteemed journal predominantly explore the enhancement of aesthetic and biological properties through the addition of titanium in composites. However, the research underscores the necessity for improvements in their microstructure and properties to meet the demands of future dental implant applications. ^40^ Moreover, a separate study within the journal investigates the impact of a 2% quaternary ammonium cavity disinfectant, emphasizing its non-cytotoxic effects on fibroblasts. Additionally, the study sheds light on how this disinfectant’s anti-inflammatory properties can stimulate the healing and repair of dental tissues. ^41^

### Citations per country

**Figure 8.**
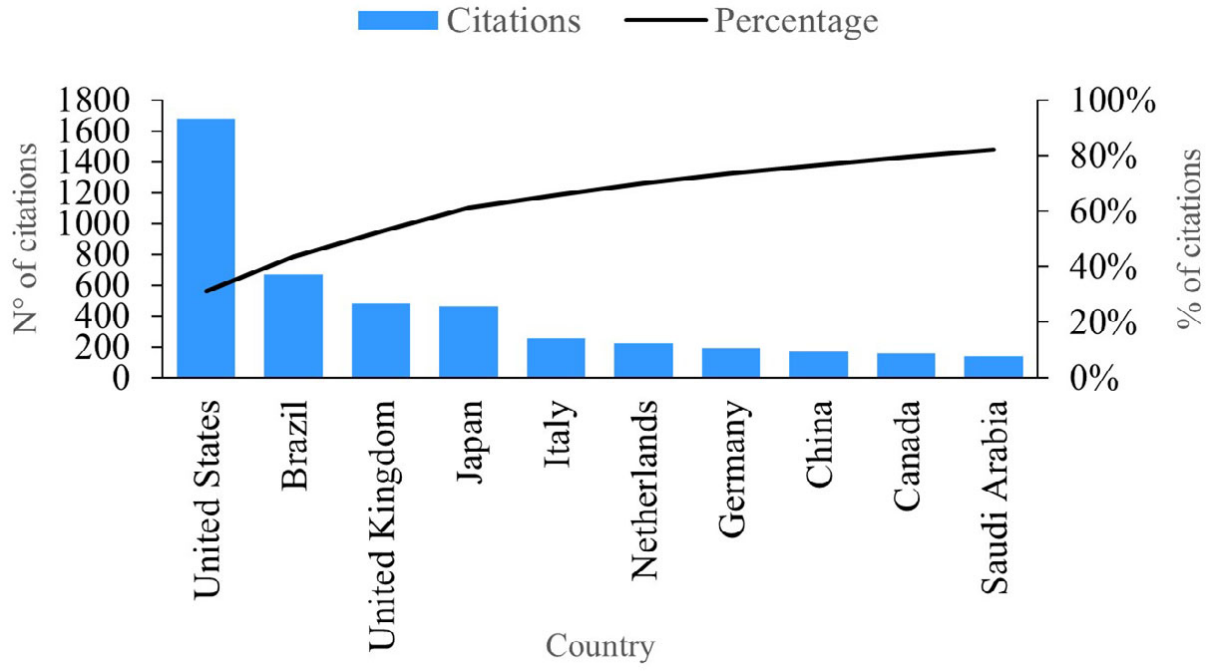
Impact per country.

In Figure 8, we observe the ten countries showcasing the highest number of citations linked to publications in the research field of PBM. Leading the pack is the United States, boasting a remarkable 1677 citations and solidifying its position as the country with the most significant impact and productivity in this domain. Notably, several publications from the United States delve into the profound impact of PBM therapy on the gene expression of postnatal dental pulp stem cells, measuring pivotal inflammatory and mineralization processes within tissues. ^42^

Illustrated in Figure 8, we observe the impact per country based on citations associated with their research publications. Notably, Brazil emerges with approximately 667 citations, establishing itself as the second most productive country in this realm (refer to Figure 5). Brazil’s publications focus on the treatment of opportunistic oral diseases associated with COVID-19 utilizing PBM and antimicrobial photodynamic therapy, resulting in noteworthy effectiveness by eliminating associated symptoms and alleviating pain. ^43^

**Figure 9.**
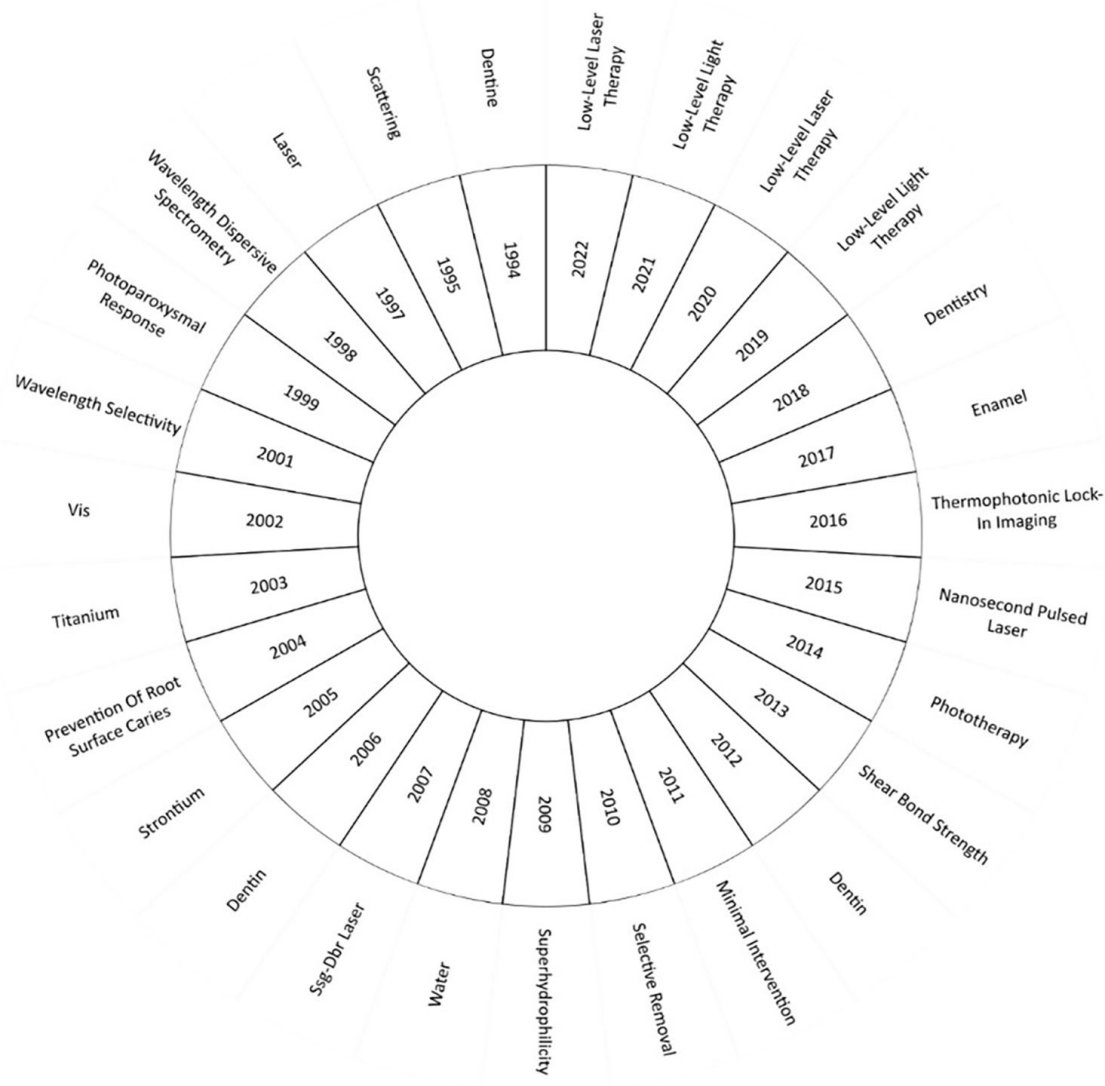
Evolution of keywords by year.

Shifting our focus to the evolution of conceptual literature on PBM, we analyze its progression. Figure 9 sheds light on the most significant keywords in research for each year. In the investigations of 1994, the concept of “dentine” (or dentin) took center stage, particularly exploring techniques to measure caries in secondary dentin. ^44^ Remarkably, this concept has retained its importance in the literature, being the most investigated term in both 2006 and 2012.

In 2014, the research field saw phototherapy at its core. For instance, a study by Ref. 13 meticulously analyzed the benefits of low-level laser therapy emphasizing its role in healing, inflammation reduction and pain management. Furthermore, as posited by Ref. 45, a single dose of LED irradiation can effectively biomodulate oxidative stress in dental pulp cells.

As we progress to the years 2019, 2020, 2021 and 2022, "low-level laser therapy" emerges as the most investigated topic. Research in this domain demonstrated the positive impact of PBM in individuals with dental implants. ^46^ However, it also shed light on a limitation concerning the efficacy of PBM in postoperative implant patients. Consequently, the authors recommend further exploration through randomized controlled trials to scrutinize various variables. ^47^

## Discussion and conclusions

### International scientific cooperation

**Figure 10.**
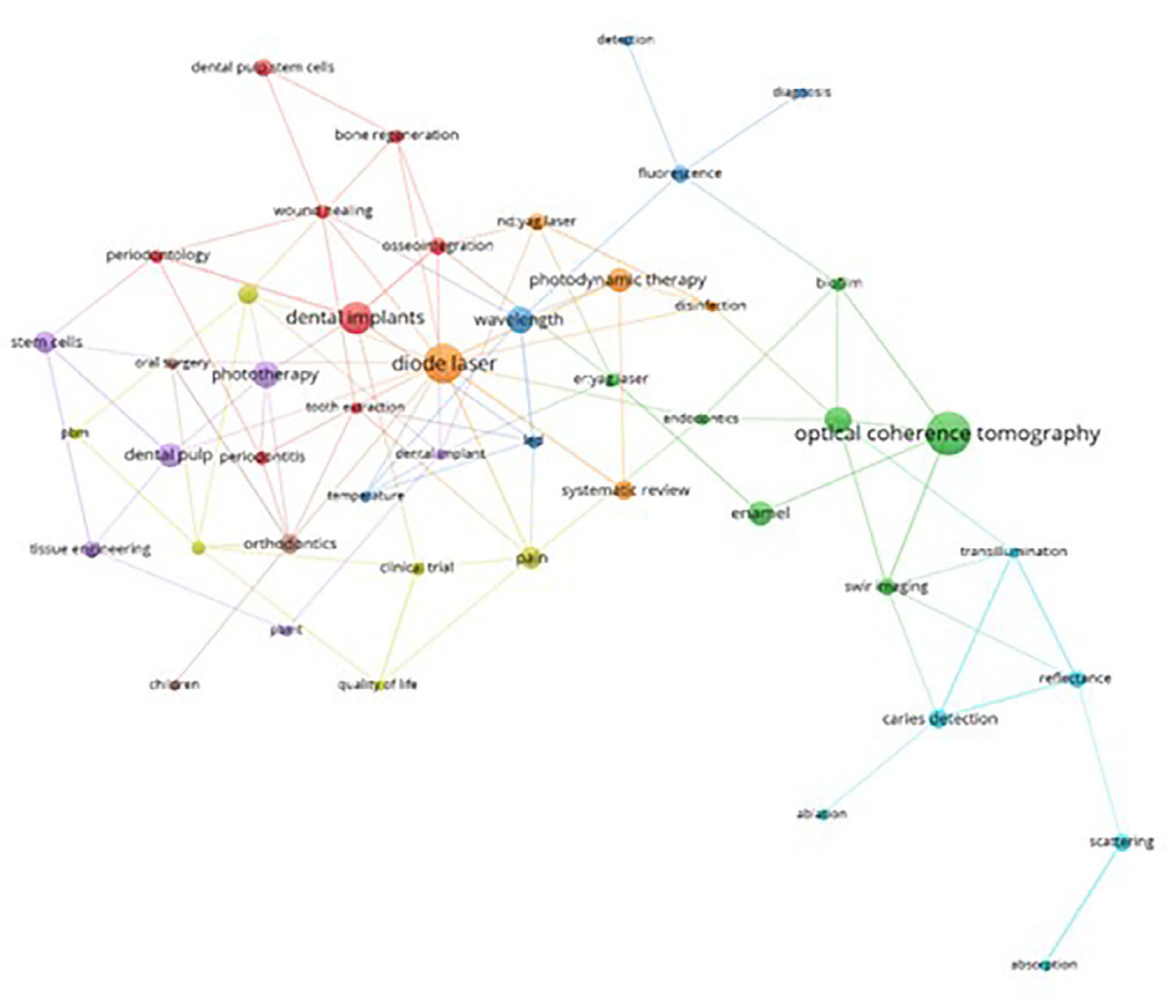
Thematic cluster grouping.

This bibliometric study facilitated an in-depth analysis of keywords associations within the scientific production on PBM. Figure 10 delineates the principal thematic cluster highlighted in green. Within this cluster, “Optical Coherence Tomography” (OCT) emerges as the central theme interlinked with other vital terms such as “Enamel”, “SWIR Imaging" (spiral images), “Er:YAG Laser,” “Biofilm,” and”Endodontics”. OCT s prominence stems from its ability to generate images within a depth range of 2 and 3 mm, allowing for a structural characterization at the enamel and dentin levels. This aids in determining the extent and progression of structural issues, pivotal for accurate diagnoses and treatment planning (Clarkson, 2014). Notably, OCT in the realm of endodontics has demonstrated superior results compared to computed microtomography, especially in visualizing empty spaces concerning apical filling. ^48^ Furthermore, it has shed light on PBM’s potential application in cell regeneration within endodontic treatments. ^49^

The orange thematic cluster “Diode Laser” stands out as the second most relevant in the study of PBM. It is intricately associated with keywords like “Photodynamic Therapy,” “Nd:YAG Laser,” and “Disinfection.” Photodynamic therapy, an alternative antibacterial therapeutic modality, has underscored the efficacy of diode lasers in enhancing periodontal clinical parameters after implementing a comprehensive oral disinfection protocol in nonsurgical procedures. ^50^ These lasers have showcased bactericidal prowess and promising outcomes in the treatment of infectious diseases. ^51^ Blue and blue–violet diode lasers have gained widespread application in dentistry, serving as effective devices in clinical treatments across dental surgery, endodontics, oral surgery, orthodontics, periodontics, and dental aesthetics. Moreover, they find utility in disinfection and PBM. ^52^

Within the red cluster themed around “Dental Implant”, vital keywords like “Osseointegration,” “Dental Pulp Stem Cells,” “Bone Regeneration,” “Wound Healing,” “Periodontology,” “Tooth Extraction,” and “Periodontitis” hold prominence. A majority of findings within this cluster are associated with favorable alterations in cell proliferation, particularly highlighting the potential of growth factors in fibroblasts and osteoblasts. ^53^ Studies exploring stem cells from dental pulp suggest a positive response to phototherapy. ^54^ TPBM has emerged as a subject of research, showcasing its potential in generating anti-inflammatory and analgesic effects during the bone repair process within dentistry. ^55^

In the purple cluster, focusing on “Phototherapy”, key terms such as “Dental Pulp,” “Stem cells,” “Tissue Engineering,” “PBMT,” and “Dental Implant” hold significance. These terms are associated with studies encompassing a spectrum of investigations, ranging from complementary therapies to regenerative endodontic treatments, ^56^ as well as the regeneration of dental tissues and stem cells. ^57^^–^^59^

**Figure 11.**
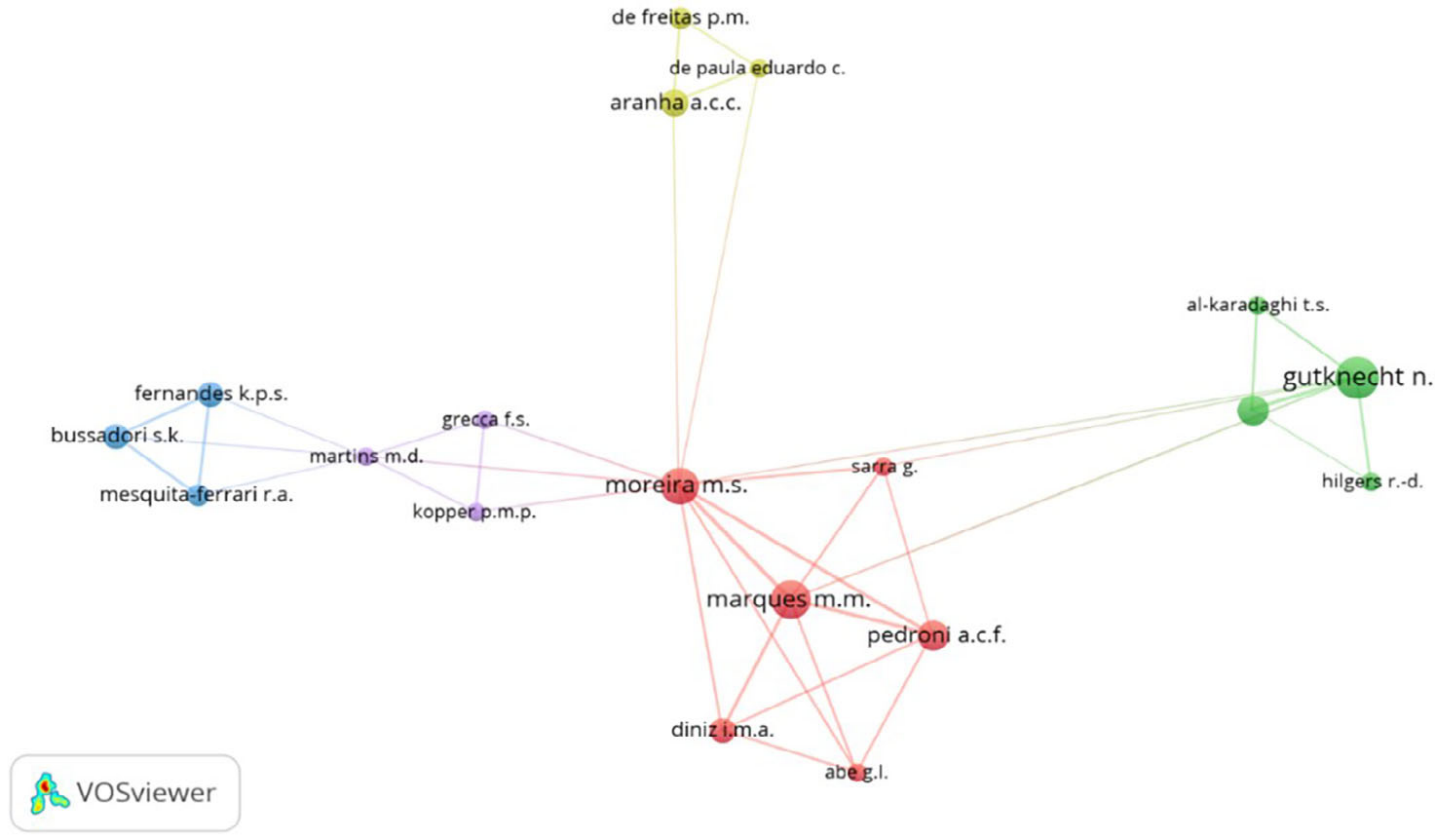
Coauthorship clustering.

Figure 11 presents a network highlighting coauthorship or associativity among authors contributing to the scientific production on PBM. The dominant cluster, depicted in red, features notable authors like Moreira M, Marques M, Pedroni A, Sarra G, Diniz I and Abe G. Their contributions have shed light on how PBM can be effectively employed in cell biology, owing to its cell therapy and tissue engineering properties. ^60^

Within the green cluster, notable authors such as Gutknecht N, Al - Karadaghi T, and Hilgers R are featured. Their research has demonstrated that dual wavelength lasers neither induce harmful thermal changes nor negatively impact sterilization of the root canal or the removal of the smear layer during endodontic treatment. ^61^

Moving to the clusters’ delineation, the yellow cluster comprises De Freitas P, De Paula Eduardo C and Aranha A, while the purple cluster involves Martins M, Grecca F and Kopper P. Finally, the blue cluster highlights the contributions of authors Fernandes K, Bussadori S and Mesquita - Ferrari R, who have significantly enriched the body of knowledge surrounding PBM.

**Figure 12.**
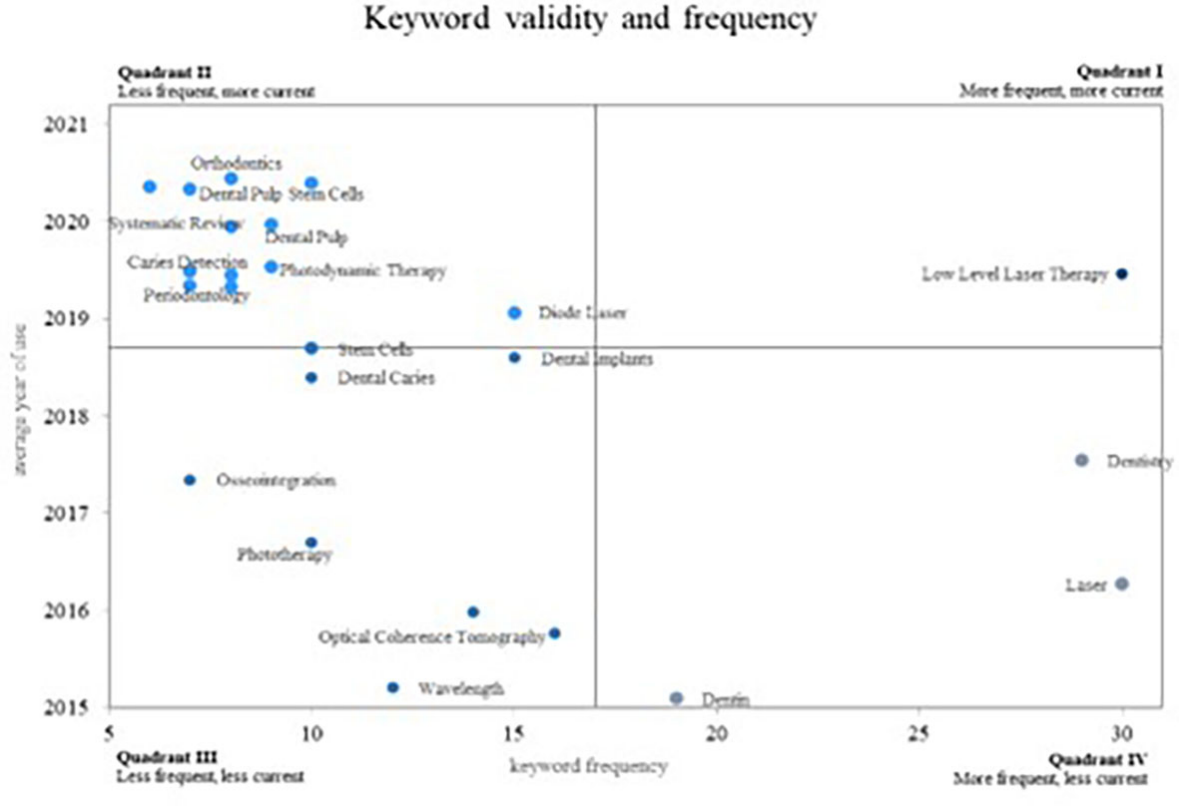
Validity and frequency of keywords.

Concerning the keyword analysis of the scientific activity associated with PBM, Figure 12 employs a Cartesian plane to juxtapose the frequency of keyword usage with the average year of use. This representation delineates four quadrants, each offering distinct insights. Quadrant I encompasses the most frequent used and current keywords, Quadrant II features the least frequent yet most current keywords, Quadrant III encompasses both the least frequent and the least current keywords, and Quadrant IV comprises the most frequently used keywords, although they appear less frequently in the current scientific literature.

In Quadrant IV, the most frequently occurring yet less current words are positioned indicating diminishing relevance in the research field. Here, we find two significant concepts: “Dentistry,” crucial for demonstrating the utility of fibre tips in various dental laser applications, ^62^ and “Laser” and “Dentin”, where authors emphasize a less invasive technique for selective removing dental caries through laser due to its absorption by organic matter. ^63^

Quadrant III comprises less frequent and less current concepts in the scientific literature. These concepts are likely to lose prominence in future PBM research: “Osseointegration,” “Phototherapy,” “Optical Coherence Tomography,” “Wavelength,” “Dental Caries,” and “Dental Implants.”

Quadrant II holds the most current keywords but with lower research frequency, signifying emerging trends: “Dental Pulp Stem Cells,” “Dental Pulp,” “Photodynamic Therapy,” “Diode laser,” “Caries detection,” “Stem cells,” and “Periodontology.” Additional concepts encompass “Orthodontics,” where authors have evaluated the outcomes of periodontal laser therapy in controlling inflammation after orthodontic tooth movement, ^64^ and “Laser therapy,” demonstrating that PBM via laser irradiation enhances bone integration. ^65^

Quadrant I houses the most frequent and current concepts, considered to be growing concepts and highly relevant within the scientific community. This quadrant exclusively features the keyword “low-level laser therapy,” proven to yield a higher success rate in pulpotomy procedures for primary molars. ^66^

**Figure 13.**
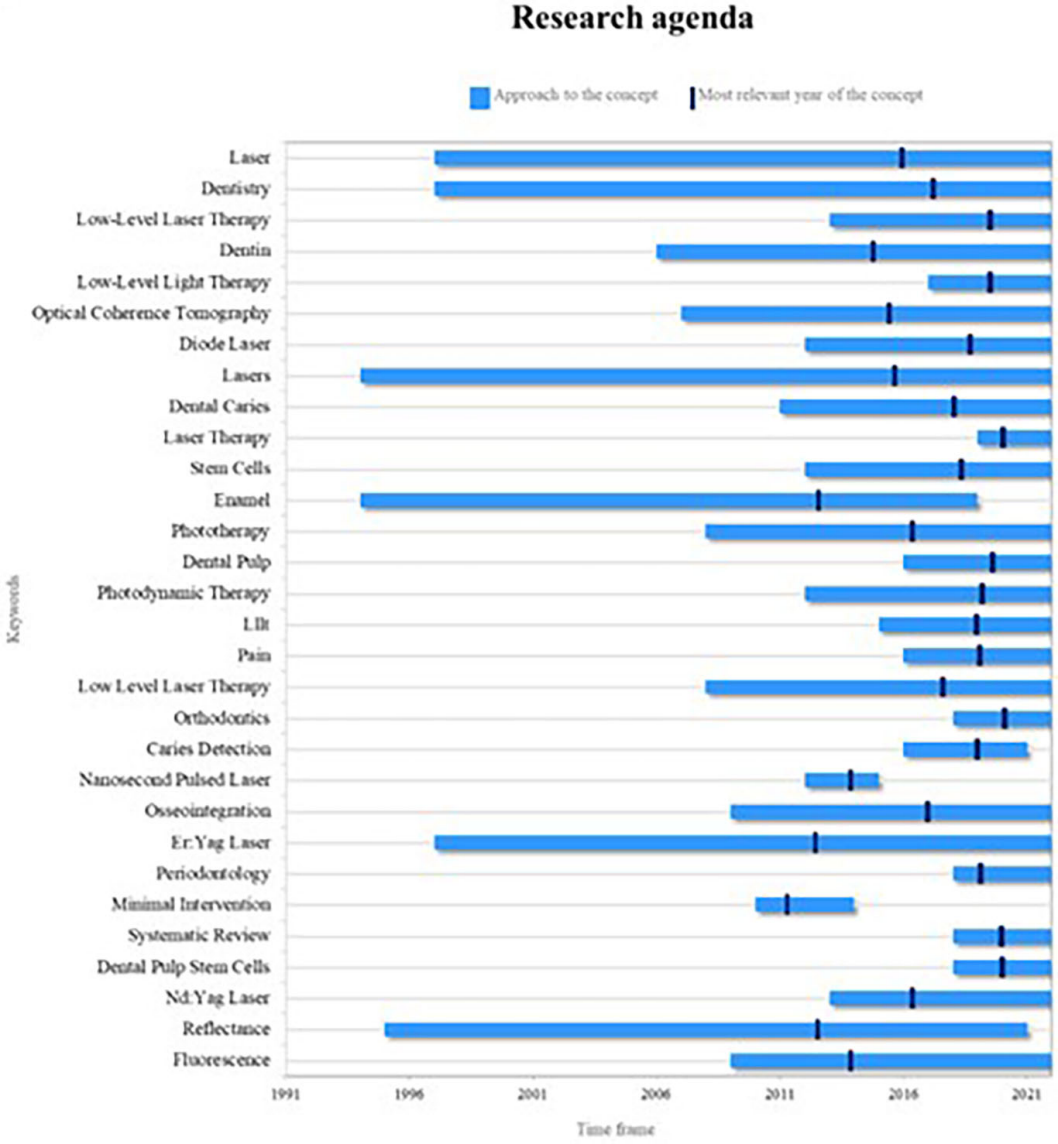
Research agenda.

The discerned trends in keywords through this bibliometric analysis enable the determination of the research agenda. This agenda serves as a foundational input for future researchers, encouraging them to delve into recent and pertinent topics, thereby ensuring the scientific community is nourished with cutting-edge information ( Figure 13).

The primary themes arising from PBM are presently significant, encompassing concepts like lasers and the overarching analysis of dentistry. These have been extensively explored over a substantial timeframe, contributing to a wealth of information within the scientific realm.

However, among these key concepts, some have recently emerged in research but have now become foundational. This indicates their potential to assume central positions in the forthcoming landscape of PBM research. Notable among these are low-level laser therapies or low-level light therapies, as elaborated earlier, and diode lasers, which have gained prominence as widely used technologies in dentistry in recent years.

Another emergent concept steering future research is orthodontics, intimately connected to laser therapies, forming a pivotal axis within PBM as expounded earlier, and in association with endodontic treatment.

As depicted in Figure 13, not all key concepts hold equal significance for future research. For instance, enamel analysis, a subject addressed in the mid-1990s with a surge in 2015, has since witnessed a decline in related studies, indicating a diminishing trend post-2019. Similarly concepts like caries detection, pulsed nanosecond lasers, and minimal interventions lack substantia elaboration from authors, thereby diminishing their relevance in future research directions.

In conclusion, research in PBM has undergone exponential growth in recent years, showing a pronounced inclination towards low-level laser therapy and the utilization of emerging technologies such as laser diodes in dentistry. This evolution is evident in the thematic progression, shifting from initial focal points like “dentine” and “caries” to broader and intricate treatments, particularly in areas like tissue regeneration and cell therapy involving dental pulp stem cells. Additionally, several burgeoning research terms such as “diode laser,” “caries detection,” “orthodontics,” and “low-level laser therapy” have emerged and are anticipated to assume pivotal roles in future PBM studies.

Hence, the research agenda for PBM is characterized by the integration of diverse study areas, melding technologies like optical coherence tomography (OCT) with the analysis of dental tissues like enamel and dentin. Concurrently, a decline in the relevance of previously pivotal topics such as “osseointegration,” “phototherapy,” and “dental implants” has been observed, indicating a shift in research priorities. This bibliometric review serves as an invaluable compass for prospective researchers and professionals keen on PBM, offering a clear vision of emerging trends and most pertinent subjects in this dynamically evolving field.

## Data Availability

Zenodo. Trends in Photobiomodulation in Dentistry between 2018 and 2022: Advances and Investigative Agenda. DOI: https://doi.org/10.5281/zenodo.8411713. ^67^ Data are available under the terms of the Creative Commons Attribution 4.0 International license (CC-BY 4.0). Zenodo. Trends in Photobiomodulation in Dentistry between 2018 and 2022: Advances and Investigative Agenda. PRISMA checklist. DOI: https://doi.org/10.5281/zenodo.8411713. ^67^ Data are available under the terms of the Creative Commons Attribution 4.0 International license (CC-BY 4.0).
