# Supplementary material for: A systematic review of trends in photobiomodulation in dentistry between 2018 and 2022: advances and investigative agenda
Source: F1000Res. 2023 Dec 28;12:1415. Originally published 2023 Oct 27. [Version 2] doi: 10.12688/f1000research.140950.2 (PMC10823425; doi:10.12688/f1000research.140950.2)
Supplement: Peer review history

# Reviewer report: Reviewer response for version 2

**DOI:** 10.5256/f1000research.159885.r233989

**Published:** 2024-02-26

**Authors:** Idalia Rodríguez Delgado, Norma Cruz Fierro

**Affiliations:** 1 Universidad Autónoma de Nuevo León, Nuevo León, Mexico; 2 Universidad Autonoma de Nuevo Leon, San Nicolás de los Garza, Nuevo Leon, Mexico

I confirm receiving the new version of the article with the adjustments made which are appropriate and pertinent. 

 Only figures 12 and 13 are not clear, the words are not clear, the other figures are very good.

Is the work clearly and accurately presented and does it cite the current literature?

Yes

If applicable, is the statistical analysis and its interpretation appropriate?

Not applicable

Are all the source data underlying the results available to ensure full reproducibility?

Yes

Is the study design appropriate and is the work technically sound?

Partly

Are the conclusions drawn adequately supported by the results?

Yes

Are sufficient details of methods and analysis provided to allow replication by others?

Yes

Reviewer Expertise:

NA

We confirm that we have read this submission and believe that we have an appropriate level of expertise to confirm that it is of an acceptable scientific standard.

# Reviewer report: Reviewer response for version 2

**DOI:** 10.5256/f1000research.159885.r233990

**Published:** 2024-01-27

**Authors:** Brenda Yuliana Herrera Serna

**Affiliations:** 1 Universidad Autonoma de Manizales, Manizales, Caldas, Colombia

Thanks to the authors for their availability and openness to suggestions. The study is proving to be useful in clinical practice, which should be the ultimate goal of research. I have no further comments.

Is the work clearly and accurately presented and does it cite the current literature?

Yes

If applicable, is the statistical analysis and its interpretation appropriate?

Not applicable

Are all the source data underlying the results available to ensure full reproducibility?

Yes

Is the study design appropriate and is the work technically sound?

No

Are the conclusions drawn adequately supported by the results?

Yes

Are sufficient details of methods and analysis provided to allow replication by others?

Partly

Reviewer Expertise:

Epidemiological and evidence analysis studies.

I confirm that I have read this submission and believe that I have an appropriate level of expertise to confirm that it is of an acceptable scientific standard.

# Reviewer report: Reviewer response for version 1

**DOI:** 10.5256/f1000research.154358.r219906

**Published:** 2023-11-16

**Authors:** Idalia Rodríguez Delgado, Norma Cruz Fierro

**Affiliations:** 1 Universidad Autónoma de Nuevo León, Nuevo León, Mexico; 2 Universidad Autonoma de Nuevo Leon, San Nicolás de los Garza, Nuevo Leon, Mexico

As part of the advances in dentistry, laser therapy is currently being used to improve the results of different types of dental treatments, hence the importance of having an analysis of the uses of Photobiomodulation (PBM).

 Below are some observations that were found in the article, with the purpose that these contributions serve for a better quality of the article.

 During the development of the writing, it is observed that a bibliometric analysis was carried out in two databases and although they mention that they used the PRISMA declaration for the exclusion criteria, it is suggested that this criterion be eliminated since it does not comply with the entire PRISMA protocol.

 In the introduction at the end only place the general objective or purpose of the study and eliminate the conclusions only place them at the end of the article.

 It is recommended to improve the quality of some of figures, since they are not clearly visible, it is suggested to include others with better quality of definition.

 Congratulations the authors of this article that demonstrates current trends in dentistry, hoping that these recommendations will be useful to improve the quality of their writing.

Is the work clearly and accurately presented and does it cite the current literature?

Yes

If applicable, is the statistical analysis and its interpretation appropriate?

Not applicable

Are all the source data underlying the results available to ensure full reproducibility?

Yes

Is the study design appropriate and is the work technically sound?

Partly

Are the conclusions drawn adequately supported by the results?

Yes

Are sufficient details of methods and analysis provided to allow replication by others?

Yes

Reviewer Expertise:

Odontology

We confirm that we have read this submission and believe that we have an appropriate level of expertise to confirm that it is of an acceptable scientific standard, however we have significant reservations, as outlined above.

# Response

**Published:** 2023-11-29

**Authors:** JHOANY ALEJANDRO VALENCIA ARIAS

We appreciate the review of our manuscript titled " **Trends in photobiomodulation in dentistry between 2018 and 2022: advances and investigative agenda **" by the reviewers. We value the detailed and constructive feedback they have provided. We would like to inform you that we are in the process of preparing detailed responses to each reviewer, and we will be sending them separately in response to their valuable insights. We are committed to addressing each of the points raised and improving the quality and clarity of our work based on the received feedback.

 Comments of Reviewer 3

 During the development of the writing, it is observed that a bibliometric analysis was carried out in two databases and although they mention that they used the PRISMA declaration for the exclusion criteria, it is suggested that this criterion be eliminated since it does not comply with the entire PRISMA protocol.

 In the introduction at the end only place the general objective or purpose of the study and eliminate the conclusions only place them at the end of the article.

 It is recommended to improve the quality of some of figures, since they are not clearly visible, it is suggested to include others with better quality of definition.

 Response to Reviewer 3

 Thank you for your thoughtful feedback on our manuscript. We appreciate your keen observations and have taken them into careful consideration. Regarding the bibliometric analysis, we acknowledge the limitations of conducting it in only two databases. However, due to resource constraints and the nature of our study, expanding the database coverage might not be feasible. We have revised the manuscript to eliminate the mention of using the PRISMA declaration for exclusion criteria, recognizing that our approach does not fully adhere to the complete PRISMA protocol.

 In the introduction, we have now revised it to conclude with only the general objective or purpose of the study, as suggested. Additionally, we have moved the conclusions to the end of the article to enhance the overall structure.

 We appreciate your comment on the figures and have endeavored to improve their quality. High-definition images have been included to enhance visibility and clarity.

 Once again, we thank you for your valuable insights, which have undoubtedly contributed to the refinement of our manuscript.

# Reviewer report: Reviewer response for version 1

**DOI:** 10.5256/f1000research.154358.r219907

**Published:** 2023-11-09

**Authors:** Brenda Yuliana Herrera Serna

**Affiliations:** 1 Universidad Autonoma de Manizales, Manizales, Caldas, Colombia

The authors are thanked for synthesising evidence of a development that should have wider application. The major concern is with respect to methodology. They announce a literature review but combine elements of what is intended to be a systematic review. Something in the middle is a scoping review. 

 In effect, the methodology reflects the characteristics of an analysis that would not meet the specifications of a full systematic review. To adhere to a bibliometric analysis it is suggested to extend the search to more than two databases including, among others, grey literature.

 I suggest reviewing the PRISMA indications for scoping and for protocol. I understand the intention of the bibliometric analysis, but they may still be useful. http://www.prisma-statement.org/Extensions/ScopingReviews

Is the work clearly and accurately presented and does it cite the current literature?

Yes

If applicable, is the statistical analysis and its interpretation appropriate?

Not applicable

Are all the source data underlying the results available to ensure full reproducibility?

Yes

Is the study design appropriate and is the work technically sound?

No

Are the conclusions drawn adequately supported by the results?

Yes

Are sufficient details of methods and analysis provided to allow replication by others?

Partly

Reviewer Expertise:

Epidemiological and evidence analysis studies.

I confirm that I have read this submission and believe that I have an appropriate level of expertise to confirm that it is of an acceptable scientific standard, however I have significant reservations, as outlined above.

# Response

**Published:** 2023-11-29

**Authors:** JHOANY ALEJANDRO VALENCIA ARIAS

We appreciate the review of our manuscript titled " **Trends in photobiomodulation in dentistry between 2018 and 2022: advances and investigative agenda **" by the reviewers. We value the detailed and constructive feedback they have provided. We would like to inform you that we are in the process of preparing detailed responses to each reviewer, and we will be sending them separately in response to their valuable insights. We are committed to addressing each of the points raised and improving the quality and clarity of our work based on the received feedback.

 Comments of Reviewer 2

 The authors are thanked for synthesising evidence of a development that should have wider application. The major concern is with respect to methodology. They announce a literature review but combine elements of what is intended to be a systematic review. Something in the middle is a scoping review.

 In effect, the methodology reflects the characteristics of an analysis that would not meet the specifications of a full systematic review. To adhere to a bibliometric analysis it is suggested to extend the search to more than two databases including, among others, grey literature.

 I suggest reviewing the PRISMA indications for scoping and for protocol. I understand the intention of the bibliometric analysis, but they may still be useful. http://www.prisma- statement.org/Extensions/ScopingReviews

 Response to Reviewer 2

 We extend our gratitude to the reviewer for acknowledging our efforts in synthesizing evidence for a development with broader applications. The concern regarding the methodology has been duly acknowledged. The comment rightly identified the amalgamation of elements that, despite being declared as a literature review, appeared more aligned with a scoping review, indicating a deviation from the intended systematic approach. In response, we adjusted the methodology to ensure closer alignment with the specifications of a comprehensive systematic review. However, expanding the search to include more than two databases, as suggested, was not feasible, as detailed in the discussion. Such an expansion would have significantly altered all obtained results. It's important to note that all text based on the PRISMA protocol has been removed, as highlighted in the discussion. The suggestion to review the PRISMA indications for scoping and protocols was carefully considered, and our approach was reassessed accordingly.

# Reviewer report: Reviewer response for version 1

**DOI:** 10.5256/f1000research.154358.r219905

**Published:** 2023-11-03

**Authors:** Herney Garcia-Perdomo

**Affiliations:** 1 Universidad del Valle, Cali, Valle del Cauca, Colombia

Dear Authors

 Congratulations on this important effort to analyze the trends in the use of PBM in dentistry between 2018 and 2022.

 I would like to comment about specific points, and I hope these will improve the quality of your manuscript. 

1. This is not a systematic review, this is a bibliometric analysis or study. Therefore, please change any description throughout the manuscript. 
2. The final part of the introduction must describe the main objective of the study. Consequently, please, state only the main one. 
3. Delete any conclusion from the introduction section.
4. Describe in the limitation section that the bibliometric analysis was limited to Scopus. 
5. Delete the methodological design section. It is already described in the methods section
6. Delete that this is a quantitative and qualitative bibliometric analysis. Leave it as: bibliometric study or analysis.
7. Figures do not see correctly. Add high-quality images
8. Add a strengths and limitations section.
9. As this is not a systematic review, the way to write is not associated with PRISMA, I suggest deleting it.

Hope this will help you to improve the quality of your important article.

 Thank you.

Is the work clearly and accurately presented and does it cite the current literature?

Yes

If applicable, is the statistical analysis and its interpretation appropriate?

Not applicable

Are all the source data underlying the results available to ensure full reproducibility?

Yes

Is the study design appropriate and is the work technically sound?

Yes

Are the conclusions drawn adequately supported by the results?

Yes

Are sufficient details of methods and analysis provided to allow replication by others?

Yes

Reviewer Expertise:

Evidence synthesis; clinical epidemiology; education; urologic oncology

I confirm that I have read this submission and believe that I have an appropriate level of expertise to confirm that it is of an acceptable scientific standard, however I have significant reservations, as outlined above.

# Response

**Published:** 2023-11-29

**Authors:** JHOANY ALEJANDRO VALENCIA ARIAS

We appreciate the review of our manuscript titled " **Trends in photobiomodulation in dentistry between 2018 and 2022: advances and investigative agenda **" by the reviewers. We value the detailed and constructive feedback they have provided. We would like to inform you that we are in the process of preparing detailed responses to each reviewer, and we will be sending them separately in response to their valuable insights. We are committed to addressing each of the points raised and improving the quality and clarity of our work based on the received feedback.

 Comments of Reviewer 1

1. This is not a systematic review, this is a bibliometric analysis or study. Therefore, please change any description throughout the manuscript.
2. The final part of the introduction must describe the main objective of the study. Consequently, please, state only the main one.
3. Delete any conclusion from the introduction section.
4. Describe in the limitation section that the bibliometric analysis was limited to Scopus.
5. Delete the methodological design section. It is already described in the methods section.
6. Delete that this is a quantitative and qualitative bibliometric analysis. Leave it as: bibliometric study or analysis.
7. Figures do not see correctly. Add high-quality images.
8. Add a strengths and limitations section.
9. As this is not a systematic review, the way to write is not associated with PRISMA, I suggest deleting it.

Response to Reviewer 1

 We appreciate your meticulous review of our manuscript. Following your feedback, we have clarified the study's nature as a bibliometric analysis rather than a systematic review, and we've revised the introduction to succinctly state the primary objective. Any conclusions in the introduction have been removed, and the limitations section now specifies that the bibliometric analysis was confined to Scopus and Web of Science. The redundant methodological design section has been deleted, and we've adjusted the description to label the study as a bibliometric analysis without the quantitative and qualitative distinction. Figures have been replaced with higher-quality images, and we've incorporated specific sections on strengths and limitations. Lastly, any association with

 PRISMA has been removed, aligning with the study's nature. We value your insightful comments, which have significantly enhanced the clarity and quality of our article.
